# Collective memory and social imaginaries of the epidemic situation in COVID-19—based on the qualitative research of college students in Wuhan, China

**DOI:** 10.3389/fpsyg.2022.998121

**Published:** 2022-09-23

**Authors:** Renqi Luo, Weiyi Feng, Yuan Xu

**Affiliations:** ^1^School of Media and Communication, Shanghai Jiao Tong University, Shanghai, China; ^2^School of Humanities and New Media, Yangtze University, Jingzhou, China; ^3^School of Media and Communication, Wuhan Textile University, Wuhan, China

**Keywords:** youth, collective memory, social imaginaries, media, national discourse

## Abstract

This study conducted in-depth interviews with 20 students from a university in Wuhan so as to obtain data regarding their collective memory at the early stage of the COVID-19 outbreak and their social imaginaries in the longitudinal dimension of time. Compared with those in other regions, interviewees from Wuhan show more fear and dissatisfaction and think that others find it difficult to empathize with their first-hand experiences. Interviewees from Wuhan are more dependent on the media. However, media use can cause problems related to redundant information and emotional impact. While one is confined to home, he/she is forced to participate in communication with family members and the topic of the body is discussed again from a new angle. Trauma leads to self-reconciliation, as facilitated by the re-examination of and reflection on one’ nomination of and reflection on n family members and the months in the wake of the COVID-19 outbreak. However, having received a collectivist education since childhood and having been guided by the discourse system created by the state media, they have since been able to shape their sense of identity and strengthen their sense of national honor.

## Introduction

The COVID-19 pandemic has persisted for more than 2 years, causing great harm to people all over the world. Being the city where COVID-19 was first discovered, Wuhan, China has attracted great attention both in the media and in academia. To control the large-scale spread of COVID-19, the Chinese government ordered the city to be shut down. This involved Wuhan cutting off all personnel movements, allowing only designated personnel to transport necessary medicines and food. Although such extreme measures negatively impact people’s lives and economic development, the Chinese government believes that the city closure policy is the safest way to defeat COVID-19. Due to insufficient initial preparations and a limited understanding of the virus, the government response at the beginning of the outbreak was very chaotic, resulting in a wave of panic and fear.

Schewe and Meredith (2004) argue that one’s personality is generally formed between the ages of 17 and 25. The critical years hypothesis indicates that during one’s “coming of age,” the common memory of major events will remain relatively stable throughout one’s lifetime. This hypothesis has been gradually built on by subsequent studies (Schuman and Corning, 2012, 2017). Young people, as represented by college students, have little memory of the SARS virus in 2003 because they were only children at that time. Thus, when faced with the outbreak of the COVID-19, which resulted in pneumonia and sometimes death, many people belonging to the younger generation were taken by surprise. Studies have shown that people’s psychological stress increased significantly in the first few months after the COVID-19 outbreak, but after several months had passed, the level of anxiety, pain, and depression began to decrease (Aknin et al., 2021). In contrast, other studies have concluded that short-term social distance caused by isolation measures may be conducive to mental health, while long-term social distance has a negative impact on mental health (Zhang et al., 2021). This is because a temporary escape from the busy workplace and increased opportunities to spend time with family may be beneficial (de Bloom et al., 2014), but, as time passes, social isolation caused by social distancing makes people experience more negative emotions (Giacco et al., 2016). Because this subject has produced mixed findings in previous studies, this study aims to establish with more clarity what sort of psychological changes the Chinese youth underwent during the 8 months of home isolation brought about by the COVID-19 pandemic.

## Literature review

### Collective memory of disaster

Halbwachs (1992) proposed that individuals acquire memory in the social environment and recall it in the social and cultural background. Subsequently, scholars put forward various views on collective memory. Wertsch and Roediger (2008) noted that collective memory is “a form of memory that transcends individuals and is shared by a group,” while Roediger and Abel (2015) similarly assert that collective memory is “a collection of events shared by a group.”

In the 1980s, memory research gradually attracted the attention of scholars. The subject has now been addressed in many studies in the fields of biology and neurocognition, social culture, literature and art, and media and technology (Brockmeier, 2010). Public disasters seem to play an important role in collective memory research, which is no surprise given that they affect many people and are typically commemorated through remembrance services. For instance, “memorial mania” (Doss, 2012) is a feature of memory research, which is related to wars, conflicts, and disasters, such as the genocide in Rwanda (Ibreck, 2010), Hurricane Katrina (Robinson, 2009) and the London bombings (Allen and Bryan, 2011).

Before the media becomes involved, people’s commemoration and remembrance practices are often based on “materiality.” In the physical scenes of tragedies and disasters, people spontaneously display artwork, sculptures, banners, and other forms of memorializing tragedies. These memorials have the ability to maintain a specific interpretation of events in the collective memory due to the physical persistence of the landscape to which they are attached (Foote, 2003) and they also help to establish a collective memory in the early days after the occurrence of a tragedy.

With websites and social media (Ebbrecht-Hartmann, 2020) devoted to the practice of collective memory and the establishment of memorials of tragic or catastrophic events, the concept of “virtual Holocaust memory” came into being. This can be understood as a specific practice of thinking about memory, potentially containing a wide variety of complex memories emphasized by different media, especially when reviewing historical trauma (Walden, 2019).

Moreover, the widespread participation of individuals in a myriad of interactions on the Internet have produced a “writable collective memory” (Ulmer, 2005). However, as a result of this phenomenon, Internet memory space can easily become a place for memory discourse competition. Hegemonic memory is usually established by an elite culture and political system, which is often then resisted and challenged by social groups. The memory process is thus a war about narration, which promotes social values and beliefs through narration (Fisher, 1984). Traditional media set up a public agenda in news practice, which is a form of “memory setting” for the audience (Kligler-Vilenchik, 2011). Repeated news reports then remodel the audience’s memory in an attempt to publicize national policies and maintain social stability. Cao et al. (2021) analyzed the mourning of Chinese netizens for Dr. Li Wenliang, for instance, and found that netizens opposed the authority of the official discourse through their expressions of sadness, relying on collective memory to form a resistance space that deviated from official norms.

Collective memory of COVID-19 has a significant group effect. Compared with the older generation, the younger generation has a higher recall rate (Mustafa et al., 2021). Edkins and Jenny (2003) has stated that trauma “always intrudes” and “cannot be completely forgotten.” After a disaster, the memory narrative of the disaster has an important impact on the construction of citizenship and the shaping of values (Hutchison, 2016). In the context of COVID-19, though, it is not clear what effect the media memory setting has on Chinese youth, especially in terms of the individual differences between Wuhan and other cities.

### Social imaginaries of disaster

Social imaginaries are defined by Taylor (2002) as “the ways people imagine their social existence, how they fit together with others, how things go on between them and their fellows, the expectations that are normally met, and the deeper normative notions and images that underlie these expectations.” Such imaginaries are “carried in images, stories and legends” (Taylor, 2004).

Dawney (2011), meanwhile, emphasized that the imaginaries in their sensory interrelation with bodies contribute to the ongoing production of imaginaries, and Stephenson (2011) proposed that new imaginaries do not appear out of thin air—they are constructed by society. Based on these claims, the concept of social imaginaries has become an important channel for continuous exploration, negotiation, and development of social groups and the environment. It also provides ideas for understanding the deep-seated ideological context of groups. Taylor (2009), for example, explored “how memory can be used as a tool in the construction and negotiation of social imaginaries of the present through the sharing of remembrances” to determine how individuals and social groups use memory to locate their relationship with social imaginaries, while Appadurai (1996) finds that the function mechanism of imaginaries in modern society is reflected in the “indoctrination” of the media in daily life that silently shapes people’s values, thus affecting their social practices.

If people use their body senses to form disaster memories, they may gradually deviate from the memory settings of the media, which makes imaginaries complex. As Callahan (2009) points out, Chinese nationalism is consumed in the reciprocal and interactive dynamics between official policy and popular feelings. Combined with the previous discussion on collective memory, we thus propose the first research question:

RQ1: What is the youth’s collective memory and social imaginaries of COVID-19 in China?

Moreover, China’s government firmly controls and regulates official and commercial social media (MacKinnon, 2008) in an attempt to stop the spread of news that deviates from their agenda. However, as the earliest witnesses of the COVID-19 pandemic, the people of Wuhan tried to produce their own news reports that opposed media propaganda, an experience that likely caused unique psychological and behavioral changes in them. Based on this, we pose our second research question:

RQ2: How do the youth use media and how are they influenced by media?

## Research methods

As mentioned in the literature review, in the past, the study of collective memory has depended on memory data obtained from the Internet. The advantages of such an approach lie in the large sample size and the rich and diversified data. However, this method is not suitable for our research. Why is this so?

First, as these “memory fragments” represent only the emotional appeal and narrative desire at a given time, they may not offer in-depth insights into historical changes. Second, it is difficult to determine the specific identity background of one’s memory on the network and impossible to link the memory with his/her personal situation and experience. Third, online memory seems to be an enormous “memory bank,” but the release of these memories depends on a strong desire to share. Thus, memory stories that are hidden because they are never publicly expressed and/or forcibly deleted must be excavated. Fourth, in addition to the appearance of collective memory, we are more concerned about how young people construct social imaginaries. How people perceive and imagine reality determines the characteristics of their social mentality more directly than reality itself. Fifth, and most importantly, the Internet in China is a large local area network, meaning that Chinese people cannot access overseas websites through normal channels. Furthermore, the Chinese government has strict control over social media speech in China, especially negative information. Therefore, the reliability of the relevant data obtained from the Internet is low.

In order to overcome these limitations, we decided to conduct our research through in-depth interviews with small samples. Although the qualitative research of small samples cannot deduce exact causal relationships in the same way that quantitative research can, it can “explicate the ways people in particular settings come to understand, account for, take action, and otherwise manage their day-to-day situations” (Miles and Huberman, 1994). It is meaningful for this new research problem, which still needs to be explored.

We recruited 20 young people in their late teens and early twenties from a university in Wuhan, China to conduct semi-structured in-depth interviews on their campus. For the first step, we interviewed six young people who lived in Wuhan, Hubei Province, other cities in Hubei Province, and other provinces during the epidemic. After that, we recruited another 14 interviewees through the recommendation of the initial six interviewees through snowball sampling, all while trying to ensure that the areas from which the participants came were reasonably balanced. We explained the research intention to each of them and promised to protect their anonymity in the research report. All interviewees signed informed consent. During each interview, as the conversation developed and hints and themes began to emerge, we supplemented the interview content with new questions. The interview period was from February 20, 2021 to March 15, 2021.

In order to test whether the qualitative data of the 20 interviewees was sufficient, we divided it into two parts. The interview data of 17 people was coded, while the data of the other three people was used to test the theoretical saturation. We found that all the topics extracted from the interview data used for testing can be summarized into the original topics. Therefore, we believe that the interview data has reached theoretical saturation, and no new interviewees need be recruited.

All 20 interviews were completed in Chinese, with each lasting a minimum of 40 min, a maximum of 123 min, and an average of 80 min. In the interview outline, the open questions mainly focus on information, pressures, family, feelings, and reflections related to the COVID-19 pandemic. During the interviews, we adjusted the questions as needed depending on the answers given by the interviewees. To establish a foundation of mutual trust and to create a positive interview atmosphere, we used general questions to “break the ice” at the beginning of each interview. The basic information of interviewees is shown in Table 1.

**TABLE 1:** Summary of basic information of interviewees.

Name code	Gender	Min	Hometown	Age
ZRJ	Female	42	Wuhan, Hubei	20
LYT	Female	45	Wuhan, Hubei	24
WZX	Male	43	Wuhan, Hubei	22
YZJ	Female	40	Wuhan, Hubei	22
LYQ	Female	41	Wuhan, Hubei	23
WJX	Male	123	Wuhan, Hubei	24
YJ	Female	48	Wuhan, Hubei	22
YLX	Female	49	Suizhou, Hubei	22
YH	Female	53	Chibi, Hubei	19
WM	Male	48	Enshi, Hubei	20
XYM	Male	58	Enshi, Hubei	24
CS	Female	41	Huanggang, Hubei	23
LYQ	Male	45	Heze, Shandong	24
ZXF	Female	61	Weifang, Shandong	21
ST	Male	45	Qingdao, Shandong	23
ZNH	Male	50	Zibo, Shandong	21
WXZ	Male	47	Heze, Shandong	23
LSW	Male	40	Handan, Hebei	23
XZY	Female	41	Yibin, Sichuan	19
LJ	Male	45	Zhumadian, Henan	23

In addition, in order to complete the triangulation, we tried to obtain the “internet memory” of the interviewees as a supplement, that is, the information, stories, and emotions they posted on social media at the beginning of the COVID-19 pandemic. Considering that the interviewees spoke at length about Weibo (a social media platform in China), we asked whether we could look at their Weibo content after each interview. We gained permission to do this from six interviewees in total, but, as mentioned earlier, the Chinese government has strict control over social media speech. Most interviewees were concerned that posting negative information would cause trouble for themselves, while some interviewees noted that they were not in the habit of expressing their emotions on social media. Therefore, we were unable to secure much text data through this method. Moreover, there seems to be no obvious systematic correlation between the content posted on the interviewees’ Weibo accounts and the opinions they expressed in the interviews, so we can only treat their Weibo content as a supplement to their interviews. This serves to underline that information obtained from social media channels is insufficient, thus further proving that we mainly rely on in-depth interviews to obtain data that is scientific and feasible.

## Findings

### Do not persuade others to be kind without suffering

When the epidemic broke out, XYM was residing in the mountains of Enshi, Hubei Province, where traffic was relatively blocked. XYM’s family were still buying some goods for the Lunar New Year and did not have a clear sense of the danger of the outbreak in Wuhan, 500 kilometers away. He joked, “Without looking at Weibo, I wouldn’t have known what happened at all, and the villagers feel that they have little to do with themselves.” He felt that it was the same as an ordinary long holiday, but ultimately this “holiday” stretched on for a prolonged period because of the unexpected epidemic. They were unable to appreciate the seriousness of the epidemic without access to the media. For geographical reasons, his life is not boring. He enjoys picking flowers in the courtyard in his spare time, working in the fields, and sometimes turns to craftsmanship to pass the hours. He responded with a trace of guilt: “When I saw volunteers rushing to help and donate resources on the Internet, I felt that everyone was actually suffering. Although it didn’t affect me, I had a good time here, which seemed a little unreasonable.”

There is also XZY from Yibin, Sichuan, who has similar ideas. “Lying on the sofa has a feeling of quiet and good years. After waking up, the weather gets better. When I hear insects, I feel that spring and summer are coming.” She knew that there were dangers, but when she opened her bleary eyes after a nap, she saw her family resting next to her and her cat dozing in its basket. She also felt that the light was good and the world was safe. A total of nine interviewees like XYM and XZY who do not live in Wuhan said that although they were able to obtain a lot of information about COVID-19 through the media, because neither they nor the people around them personally felt the harm caused by the epidemic, their mood was not greatly affected, even though they felt guilty about it.

However, the interviewees suffering from the epidemic in Wuhan is quite different. People know nothing about the virus, including the causes and consequences, and there is no specific medicine. However, people around them are constantly infected, and the city is in an emergency state of blockade. This is a tragic reality faced by the people of Wuhan. “During that time, I felt exhausted, and the world was destroyed…… I was afraid of losing my beloved ones at that time.” WJX lives in Wuhan and had four relatives who were infected, one after another, during the epidemic, for all of whom it was hard to find a hospital bed. He felt powerless and was most afraid of suddenly receiving the news that one of his beloved ones had died. Every day he called his relatives—even if they did not say a word, as long as the phone could be connected, he felt relieved. However, he had to wait 2 months to speak on the phone with his mother. Her physical condition was so critical that she needed a ventilator to survive. It was therefore extremely difficult for her to unlock her mobile phone screen. “I dream every day about pictures from my childhood with my mother. Like a merry-go-round, I hope to dream about my mother, but I’m afraid it’s a bad omen.”

Given that the pandemic had a varied impact depending on the geographical location, different populations may have had limited or less empathy than their compatriots residing in areas seriously impacted by the epidemic. Although some situations can be experienced second-hand through news reports, individuals who have not directly experienced an epidemic crisis will generally have a lower degree of empathy. LYQ from Wuhan went back to college after the epidemic stabilized and told her roommates what she had suffered from the epidemic. Although she was comforted by them, she still thought, “In fact, I can see that they are actually unable to understand why we are so excited, probably because they really have not experienced such a situation.” When Wuhan was closed, she was concerned that the government would abandon the people living in the city. Like others, her family wanted to drive away and escape the city, much like a scene from a sci-fi movie where destruction is imminent. LYT and YJ, who live in Wuhan, also felt that the end was coming and once considered plans to escape the city.

The closer people get to the vortex, though, the more closely they pay attention to the event by using media, thus affecting the corresponding media behavior and the ability to distinguish fact from fiction. Five interviewees from Wuhan did a lot of unintelligible things blindly and anxiously. YZJ had a high fever at that time and the hospital was saturated. She chose to believe in various remedies presented to her on Weibo, and treated herself as an experimental vessel for independent medical treatment, such as eating oxidized garlic slices, using salt water and vinegar for disinfection, and drinking Huoxiang Zhengqi water to suppress the virus, but her allergy to alcohol caused a series of subsequent complications. LYQ felt the silence of the Wuhan media during the early stages of the epidemic. She had a certain level of resistance to the official media from the start and stated that she would be more inclined to believe in network information, from passive reception to active search, so as to form her own understanding of the epidemic. On New Year’s Eve, what she saw on the Internet was an overcrowded hospital, the emotional breakdown of medical staff, and the patients’ fear of diseases, but, in stark contrast, the Spring Festival gala on TV showed a peaceful scene. She expressed her dissatisfaction on Weibo and told us that she thought “it was difficult to control myself at that time.” Among the six interviewees who authorized us to access their Weibo accounts, she is the only one who openly expresses herself on Weibo.

In the eyes of others, some unrealistic thoughts, behaviors, and grievances shrouded in great panic seem to hard to understand. As LYT said, “It is said on the Internet that closing the city means being responsible for people outside, and people inside the city start a random pattern of fate (Only God knows who and when will be infected with the virus) … If they were not in Wuhan at that time, it is hard to realize that kind of huge panic now!” YJ, who was also in Wuhan, expressed a similar view. She felt that the anti-epidemic exhibition held by the government in Wuhan was for outsiders because “those photos or videos are imprinted on the memory of Wuhan people,” thus suggesting that outsiders cannot fully understand the suffering of the people of Wuhan. LYQ summed up why others can’t understand herself with a Chinese proverb: “Do not persuade others to be kind without suffering.”

### Information comes from all directions

WX recalled that “The news of Weibo spread the fastest, and it was so complicated that my mind exploded.” Indeed, while CCTV News Weibo has responded to various rumors one after another, many aspects of the epidemic have yet to be clarified, causing public opinion to ferment in line with the chaotic process of forwarding, commenting, and praise.

Eager to secure their safety and that of their family, interviewees in the vortex of the epidemic use the Internet extremely frequently to obtain information on the development of the epidemic. “Every morning, when I turn on my mobile phone, I look at all kinds of new cases and deaths pushed by Weibo” (LYT). Media, now deeply embedded in daily life, has become the most convenient and rapid tool to obtain information. With the influx of information, channel differences and information asymmetry also have an influence on personal cognition. When there is a division between the “truth” seen with one’s own eyes and what is portrayed by the media, the psychological impact on people can be particularly severe. When the body is activated in panic, people prefer to believe that “seeing is believing.” In this way, the picture conveyed by the media is comparable to the illusion of Plato’s “Allegory of the Cave.” A total of five interviewees from Wuhan expressed this sense of division very clearly, as shown by the following remarks.

“What you see with your own eyes is really different from what you know by watching TV” (YJ).

“Every time I watch the news, I feel that the outside world is different from my world” (YLX).

“The official information is not very timely and the disclosure is not very accurate” (LYQ).

As a communication student, WJX noted that he understood that the original function of the mainstream media is to report positively and relieve social emotions in times of crisis. However, because of the negative experience of having four relatives become infected, he bluntly said that “there was much exaggerated propaganda in the positive news on TV.”

At the beginning of the epidemic, interviewees’ distrust of official media arose from government hesitation and prevarication as well as from the pull of information from other channels, especially from what the interviewees personally saw and heard in Wuhan vs. what was displayed on official media.

### Escape from the media

The bombardment of information from multiple channels leads to anxiety and burnout (Wurman, 1989). In turn, anxiety results in the temporary contraction of information processing capacity (Huang et al., 2012). In this way, the psychological burden and anxiety creates a vicious circle. At this time, some interviewees are choosing to turn off the information input channels. In the early stages of the epidemic, when information was erupting in a blowout state, CS continued to pay attention to the data changes of the epidemic. However, as the death toll increased, to avoid excessive psychological burden and post-traumatic stress disorder, she has refused to consume news related to the epidemic and no longer pays much attention to any news published on the network platform. In her opinion, paying too much attention to the life and death of others is a pointless exercise—it makes her feel sad and overwhelmed by a sense of powerlessness when presented with complicated information. The other six interviewees expressed similar views.

Internet public opinion is inevitably mixed with slander. YJ became aware through Weibo that an alumnus she knew was unfortunately infected with the virus. This alumnus spoke about her experience of being infected and the lack of medical treatment on Weibo. Consequently, her Weibo attracted the attention of tens of thousands of people. In addition to receiving positive blessings, she also received some material help. However, at the same time, some people made verbal attacks in the comments section and questioned her statements. They thought that she was taking advantage of the epidemic to gain attention—in other words, she was selling her misery. They speculated with inter-group prejudice, joked about regions, and made discriminatory remarks to her. YJ is very angry about this. “I want to do my best to protect the people I know, but for the first time, I realized the powerlessness caused by verbal violence” (YJ). Compared with the threat that viruses pose to health, verbal confrontation seems to be more frustrating. Since then, she has reduced the frequency with which she uses Weibo. Besides, she also talked about the harm caused by too much negative information on the Internet: “If you don’t surf the Internet, you will feel that life is peaceful and steady. If you surf the Internet, you will feel that you are experiencing war. People are dying all the time, probably in the street near you! Or the building next to you … I will refrain from reading these news … or I will become very sad.”

### Family and peer support

McLuhan (1994) thinks that “media is the extension of man,” connecting people’s communication and emotions. Peters (2012) raises an intriguing question: To what extent can the body remain absent from human communication? Can seemingly omnipotent media really become an extension of the body, completely replacing all bodily functions?

During the home quarantine, the body was confined to a relatively small, closed environment. they use primitive body functions to show language, expressions, actions, and other symbols to interact with their families, thus gaining support from their families. This is an unexpected gain for young students who are accustomed to using the media to communicate with their families.

XZY admitted that she had never felt the importance of family support before the epidemic, an emotional state that had become a barrier for mutual communication. However, the crisis became a hub for family relationship mitigation, enabling the resolution of conflicts with the integration of time and space. She said: “Whether it is normal or ineffective communication, we can only communicate. On the contrary, our relationship has greatly improved and there has been a leap forward in progress. I haven’t experienced the feeling of worrying about my family for a long time, but the epidemic has deepened everyone’s ability to understand each other in his/her unique way.” The other interviewees expressed similar views:

“I think family is still very important after getting along with my family for a long time. Because of the epidemic, we can spend more time with each other” (LJ).

“When I was a child, we didn’t have a lot of family atmosphere because everyone was busy … I’m very grateful for this time. This time has helped our family get to know each other” (ST).

The long holiday provides the interviewees with the opportunity to live and communicate with their families for a long time. It also means that campus couples are isolated from each other in their family homes and have no means of meeting up on campus. Even if a social network has established a space for communication with multimedia, it remains difficult to make up for the communication defects caused by “body absence.” After talking about the support from family, ST was somewhat lonely in explaining the reason for breaking up with his girlfriend. It was difficult for them to get support from each other. He said: “I made video calls with my girlfriend every day. But we didn’t know what to say through the video. We couldn’t meet each other, there was less contact and less interaction. Everyone is an emotional animal, unable to communicate feelings face to face, including contradictions, and many things cannot be explained.”

In response to the government’s call to “stay at home,” playing games for a long time was legitimized. Playing games soothes the mind and distracts people from their grief. Indeed, seven interviewees noted that they spent a relatively long time playing video games in order to kill time and numb themselves, so as to make themselves feel better, as shown by the following remarks.

“I look for classmates to play games with in the afternoon … maybe it will be 11 p.m. at the earliest after playing. There is nothing else to do. In order to relieve my mood, I should just find something to do” (ZNH).

“We played games! If we’re tired of playing this game, we’ll play another game. If you want them to stay at home as much as possible, you play games” (LJ).

“I can be locked up for nine hours for playing games. After nine hours, I rest for 15 minutes. These 15 minutes are the most annoying for me” (ZRJ).

ZNH is the initiator of the game. He will organize his classmates to form a team and complete various game tasks to gain a sense of achievement. He was very grateful to his friends for playing games with him late for the honor of the team, which made him feel that “friendship can be highlighted in this special period.” But he also felt a little guilty. He said: “It must have wasted too much time. I felt empty after playing the game, and so should they. But during that time, I was too depressed to study, so I indulged myself. I’m very grateful to my friends who accompanied me to play the game day and night, otherwise I’d be too hard.”

### A focus on health

In our opinion, based on Chinese traditional education, college students should have lofty ideals. According to the mainstream values, when young people are tired to study or work, elders will encourage them, “You are still young, it doesn’t matter if you suffer a little.”

However, four interviewees told us very clearly that after the epidemic, they were more concerned about their health and attitude toward life, rather than pursuing social-economic status.

“Because of COVID-19, I thought health was more and more important. I basically went for a walk every day … I used to stay up all night before, but now it’s not like that. Everything is based on life” (LYT).

“I used to wish I was rich, had a high quality of life, and was envied by others, but now I prefer to be an ordinary and interesting person. Be optimistic, I must be optimistic. In fact, I accept that I am an ordinary person … I think it is enough to have the opportunity to be filial to my parents” (ST).

“Even if I’m a mediocre person, I feel it’s not easy. It’s really difficult to live a smooth and stable life” (WXZ).

### National identity

No matter where young students lived and no matter how much suffering they had endured during the COVID-19 epidemic, their national identity remained extremely strong. They believe that the country is strong and wise in decision-making, although they are not optimistic about their personal future. Although some interviewees expressed a great deal of dissatisfaction at the beginning of the interview, a total of 15 interviewees agreed with the Chinese government’s anti-epidemic policy as a whole and expressed their sense of national honor.

Two years ago, ZNH mentioned to one of the researchers that he wanted to enlist, but it was always a choice made for future employment with a strong utilitarian purpose(According to the relevant laws of China, local governments should provide jobs and a series of conveniences for ex-servicemen). In this interview, he told us the reason why he wanted to enlist was changed: “My trust in the country and national pride is much stronger than before. I see that foreign countries emphasize democracy on the Internet, but the epidemic is still so serious that no country has done well. China is really people-oriented.”

As a volunteer in the community, XZY is still nervous when reading memory. She said: “It’s not easy to control the exit of the community … No one can understand you. No matter what grievances I suffer, but I’ll try my best to overcome them.” In fact, people play different roles in social groups, disperse from each other, and bear different social responsibilities. However, appropriate distance and timely cohesion are two indispensable emotional states in modern society. In this epidemic disaster, they are eager to gather, share, and be recognized, thus joining the social collective, participating in social practice, and responding to the call for epidemic prevention in their own way, whether at the front lines of the anti-epidemic battle or at home.

Interviewees from Wuhan said that although they had complaints at the beginning of the outbreak, they were extremely satisfied with the epidemic prevention policy. In particular, WJX, the interviewee who had experienced four of his relatives becoming infected, firmly stated that after experiencing unimaginable pain, “the resources of the state, the strength of the state and the support of the people … I must choose to believe and will never question again!” In fact, his family did the same. The state issued a policy that patients infected in Wuhan could be reimbursed for medical expenses and his father donated all the reimbursement to the Red Cross without hesitation. Other interviewees also expressed similar views, as shown by the following remarks.

“As a Wuhan native, I feel abandoned, but the state has chosen to protect most people” (LYQ).

“At 8:30 p.m., everyone shouted “Stay Strong Wuhan” outside. At first, I felt very childish when I heard others shouting. Later, more and more people shouted, and then I joined in. There were people playing the national anthem. In this environment, I suddenly felt, wow, the Chinese people are united, and that kind of feeling is so warm” (YJ).

“The Liberation Army came to support … I feel that Wuhan has not been abandoned, so many people came to help us … China is still very good. It doesn’t give up on every life and tries its best to treat everyone. We have trouble here, but all provinces and cities will send rescue teams, which is very touching” (LYT).

From the initial questioning to the subsequent acceptance, everyone unanimously chose to believe in the power of the country. When reconstructing social memory, it is important to note that when they perceive environmental threats and witness the efforts of the government and citizens, they are influenced by “memory setting” and collectivist education, which is finally condensed into a stronger sense of national identity.

## Conclusion and discussion

Chinese youth have different collective memories of and social imaginaries about the COVID-19 epidemic. The interviewees from Wuhan had more negative memories of the epidemic and had more fully imagined the consequences of COVID-19, while the interviewees who lived far away from Wuhan were relatively optimistic and did not think much about trauma and a series of consequences. Under the same background, the degree of psychological perception caused by different environmental states differs significantly. Most of the respondents residing far away from the vortex of the event have a limited sense of the epidemic thanks to the way in which the media presented the reality of the situation. Thus, individual emotions and associations will vary greatly depending on the environment. Behind the stories they tell is the memory practice of personal cognitive schema. For some people, the crisis perceived by others does not pose a direct conflict to their person. They look at the chaos from thousands of miles away as the activity of temporary “outsiders.”

Depending on whether or not their personal safety was threatened, different groups paid different levels of attention to COVID-19, which led to different memory intensity (Curci et al., 2001). The differences in memory intensity between groups has been explained by Bird and Viding (2014), who suggested that one’s experience of a situation affects their level of empathy. They proposed that the mechanism by which situations affect empathy involves inferring situations according to clues, which depends on personal knowledge and experience. The people of Wuhan have memories of first-hand experiences (Pillemer, 2009), whereas people in other regions, especially in other provinces, their cognition and memory of COVID-19 is actually the result of the interaction between personal experience and media agenda setting (McCombs and Shaw, 1972), which is called prosthetic memory (Landsberg, 2004). The owner of prosthetic memory can hardly distinguish which is reliable and which is full of propaganda. Zhang (2022) believes that the narrative of disaster has become a public opinion field for the power struggle between official and folk discourse in China. Official discourse intentionally reduces the significance of negative news reports, increases praise for heroes, gratitude to medical workers, and expresses a determination to overcome difficulties to guide positive public opinion. In this way, most people will understand the COVID-19 epidemic and prevention strategies according to the officially set words. People who have no or little experience of COVID-19, however, struggle to empathize with others’ first-hand experiences of it. Although they expressed some sympathy for the people of Wuhan, it was difficult for them to fully understand those who suffered major trauma.

Compared with other regions, interviewees in Wuhan were more dependent on the media at the beginning. In order to protect themselves and get official or hearsay information in time, they use the Internet very frequently. This is consistent with the findings of Loges (1994), who found that the more a person feels threatened by their social and natural environment, the stronger their dependence on the media. However, if social media is used for too long and too much information is conveyed to people through multi-channel media, they will experience anxiety and burnout (Luqman et al., 2017; Han, 2018). More interviewees chose to reduce the use of media because they were unwilling to continue to accept the sadness caused by negative news, gradually reducing the degree of media dependence. Media system dependency will change with the changes in various environments (Ball-Rokeach, 1985).

Because people are slowly emerging from the media environment and staying at home, the topic of “Face to Face” (FTF) communication and “Computer-Mediated Communication” (CMC) has been discussed again, especially the intimacy between young students and their parents, and the alienation from their lovers. In the past, researchs on CMC paid too much attention to the establishment of strangers’ relationships. In the few comparative studies with FTF, most of them only paid attention to the influence of online communication on offline relationships. In fact, people use the Internet to communicate mainly to maintain already extant relationships, especially family and friends, rather than to establish relationships with strangers (Howard et al., 2001). Valkenburg and Peter (2007) found that for intimate relationships, online communication has a positive impact on the quality of friendship. Dainton and Aylor (2002) found that CMC has a positive effect on relationship maintenance for long-distance couples. It is worth noting that the means of CMC in previous studies are relatively single, and social presence (Short et al., 1976) and the media richness (Daft and Lengel, 1983) are not as good as modern media. On the contrary, the results seem to be different from our observations. We think there may be three reasons. First, the social presence of CMC still cannot reach the level of FTF. Through rich body language and gestures, they can feel each other’s emotions by FTF. Second, getting along with each other day and night provides them with the opportunity to communicate for a long time. No matter whether they are happy or sad at the beginning, they always have to enter a harmonious state. Third, CMC can provide a sense of social support, encouragement and comfort from lovers, family and friends, but it is difficult to replace warm hugs and kind care. The observed data and theoretical explanation may provide new ideas for the relationship between CMC and FTF.

After experiencing the disaster, Chinese youth gradually reconciled with themselves, and recognized the importance of a stable life and a healthy body. In 2021, a popular online hot word in China was “躺平” (lying-flat), which means refusing to strive for upward social mobility (Gong and Liu, 2022). Lin and Gullotta (2021) thinks that it expresses young peoples with theme frustration and exhaustion. However, in our research, we found that even if young students chose to lying-flat, they didn’t feel disappointed with the country. Chinese youth, no matter what experience they encounter, believe that as long as they unite and cooperate with the government, they will surely defeat COVID-19. Touching news stories encourage them to contribute to the national cause. The experience of the epidemic has deepened their sense of national identity. On the one hand, social traumatic events may have recreated a sense of the collective “we” (Cohen et al., 2002) because the memory caused by trauma helps to shape social identity (Neisser, 1982), which leads them to have a strong sense of honor when talking about the future of the country. On the other hand, this may be related to their collectivist education and accepted memory settings. Chinese students were taught the values of unity, sharing, altruism, hard work and so on when they were in kindergarten, which had a great impact on the shaping of their collective values. The positive news output by the official media also guides people’s thoughts, making them temporarily ignore the pain, remember the positive stories and be grateful.

## Research limitations and future prospects

The qualitative data did not complete the triangulation, especially in the sense that the social media data of the interviewees had no structural correlation with the interview data, and the research data mainly depended on the in-depth interviews of small samples, without a wider survey. During the interviews, we did not investigate the socio-economic status of the interviewees’ families. Whether this affects our research results still needs to be explored. The conclusion of a new research study using qualitative research methods is often exploratory, and no clear theoretical model is built. In future research, though, we are interested in operationalizing related concepts, using larger samples for quantitative research, and establishing a model of the relationship between the physical distance of crisis centers and psychological changes, and the relationship between media use and national identity against the background of the crisis.

The Chinese government adhered to the zero-COVID policy in 2020, which received widespread support, including from the youth, and achieved good results. However, the Omicron variant in 2022 has become highly transmissible but less pathogenic (Chen and Chen, 2022). This has caused many countries in the world to give up the zero-COVID policy, and humans began to try to coexist with COVID-19. For more than 2 years, Chinese was one of the few countries in the world that always adhered to the zero-COVID policy (Normile, 2021). Strict isolation has prevented the large-scale spread of the virus, but it has also brought great challenges to people’s lives and economic development. We are unsure about what people’s attitudes are now, especially the long-term isolation testing people’s patience. In future studies, we suggest that a large-scale public survey be conducted in order to focus on the changes in people’s attitudes over time and provide reference for the formulation of public policies.

## Data availability statement

The raw data supporting the conclusions of this article will be made available by the authors, without undue reservation.

## Ethics statement

Ethical review and approval was not required for the study on human participants in accordance with the local legislation and institutional requirements. The patients/participants provided their written informed consent to participate in this study. Written informed consent was obtained from the individual(s) for the publication of any potentially identifiable images or data included in this article.

## Author contributions

RL contributed to the conception, data analysis, and manuscript writing of the study. WF contributed to the depth interviews and manuscript writing of the study. YX contributed to the literature obtaining and analysis and performed the analysis with constructive discussions of the study. All authors contributed to the article and approved the submitted version.
